# A Technical Assessment of a Commercial GFAP Lateral Flow Assay to Establish Proof-of-Concept for Use in Traumatic Brain Injury

**DOI:** 10.1007/s10571-025-01594-6

**Published:** 2025-07-12

**Authors:** Daniel P. Whitehouse, Edward J. Needham, Joshua D. Bernstock, Edoardo Gaude, Liam Barrett, Soraya Ebrahimi, David K. Menon, Virginia F. J. Newcombe

**Affiliations:** 1 Department of Medicine: Perioperative, Acute, Critical Care and Emergency Medicine (PACE) Section, University of Cambridge, Cambridge, UK; 2 Department of Clinical Neurosciences, University of Cambridge, Cambridge, UK; 3 Department of Neurosurgery, Brigham and Women’s Hospital, Harvard Medical School, Boston, MA USA; 4 Department of Neurosurgery Boston Children’s Hospital Harvard Medical School, Boston, MA USA; 5 David H. Koch Institute for Integrative Cancer Research Massachusetts Institute of Technology, Cambridge, MA USA; 6 CRUK Cambridge Institute, University of Cambridge, Cambridge, UK; 7 Upfront Diagnostics, Pockit Diagnostics Ltd, Cambridge, UK; 8 Radcliffe Department of Medicine, University of Oxford, Oxford, UK

**Keywords:** Glial fibrillary acidic protein (GFAP), Point-of-care (PoC) systems, Traumatic brain injuries (TBI), Biomarkers

## Abstract

Glial fibrillary acidic protein (GFAP) is an emerging biomarker for the detection of acute intracranial pathology following acute brain injuries such as traumatic brain injury (TBI), stroke, and hypoxic-ischaemic encephalopathy. We undertake a proof-of-concept technical assessment of a commercial lateral flow test (LFT) for the detection of GFAP [the Upfront DX LVOne GFAP lateral flow assay (LFA)], against GFAP concentrations measured using a gold-standard assay [Single Molecule Arrays (Simoa®)-based Human Neurology 4-Plex B assay] in a TBI population. The ability of the LVOne GFAP LFA for identification of samples with GFAP concentrations above the manufacturer’s reported lower limit of detection (≥ 0.2 ng/ml) was assessed, with further assessment of the association between the LVOne and a gold-standard assay made using Spearman’s rank correlation coefficient and linear-mixed-effects modelling. Of the 50 samples, 39 had serum GFAP concentrations exceeding the reported lower limit of detection, with the LVOne GFAP LFA having a 95% (95% CI: 83%, 99%) sensitivity and a 64% (95% CI: 31%, 89%) specificity for detecting a serum GFAP concentrations over this lower limit. There was a significant positive correlation (*Rho* = 0.94, *p* < 0.001) between the Quanterix Simoa® GFAP level and the LVOne semiquantitative score, with a significant positive association seen using a linear-mixed-effects model (*p* < 0.001). In conclusion, the Upfront DX LVOne GFAP LFA is sensitive for the detection of elevated serum GFAP levels, and as such, may be a useful adjunct to the care of patients with acute brain injuries in the pre-hospital setting.

**Supplementary Information:**

The online version contains supplementary material available at 10.1007/s10571-025-01594-6.

## Introduction

The astrocyte-associated intermediate filament protein glial fibrillary acidic protein (GFAP) has shown considerable promise as a blood-based biomarker for the detection of intracranial pathology following acute brain injuries (Ebner et al. 2020; Gaude et al. 2021; Kalra et al. 2021; Durrani et al. 2024). Although peaking around a day following injury, elevations in GFAP have been shown within 30 min of TBI, with these hyperacute elevations associated with the presence of traumatic lesions detected on a CT head scan (Papa et al. 2024). In this context, GFAP levels may aid clinical decision-making concerning hospital conveyance for assessment and neuroimaging, or the choice to transfer to specialised centres following TBI (Tepas et al. 2013). This is of particular relevance in resource-limited healthcare systems where access to acute neuroimaging may be limited and/or transfer times are long. Despite the theoretical benefits of blood-based biomarkers, current assay turnaround times limit the real-world utility of these technologies in clinical settings (Newcombe et al. 2023). As such, a point-of-care (PoC) test that can provide a rapid and accurate biomarker result could facilitate the seamless integration of biomarkers into existing healthcare systems and clinical workflows aiding in the transition from research to clinical environments.

Lateral flow assays (LFAs) are diagnostic tests designed to detect and quantify specific analytes in samples such as blood or other bodily fluids. These portable, user-friendly, and durable devices are often cost-effective, easy to store, and have long shelf lives, all whilst delivering rapid results for clinicians. LFAs operate via capillary action, drawing the sample through the device and across a test strip containing antibodies for target detection. If the anolyte of interest is present, it will bind to the antibodies forming a test line and a positive reading (Koczula and Gallotta 2016). Examples of commonly used LFAs include pregnancy tests and COVID-19 tests. Examples of commonly used LFAs include pregnancy tests and COVID-19 tests. Owing to both cost and practicality benefits, there is great interest in the use of LFAs in the prehospital environment for a variety of acute presentations including troponin for myocardial infarction (Bayoumy et al. 2021), d-dimer for venous thromboembolism (Geersing et al. 2010), and C-reactive protein for bacterial sepsis (Pohanka 2022).

In this analysis we investigated a novel proprietary LFA test (LFT) for the detection of GFAP, the Upfront DX LVOne GFAP LFT (https://upfrontdiagnostics.com/). The version of the device tested provides a binary “positive or negative” result based on a manufacturer-defined lower limit of detection (0.2 ng/mL), alongside a semiquantitative assessment where the colorimetric intensity of the test line is compared to an external colorimetric scorecard developed by the manufacturer, with a score assigned ranging from 1 to 12. In the only prior published assessment of the device, assessing the use of the LFA in patients with suspected stroke, the LVOne GFAP LFA demonstrated a significant positive correlation (*Rho* = 0.86) between test results and plasma GFAP concentrations measured using a hospital-based PoC test platform (Abbott iSTAT Alinity, TBI plasma cartridge) (Gaude et al. 2025). Furthermore, when used in combination with a d-dimer assay and clinical assessment the LVOne GFAP demonstrated good sensitivity (75%) and specificity of (92%) for the detection of large vessel occlusion stoke (Gaude et al. 2025). However, at the time of writing, the LVOne has only been evaluated in a single disease state, with no previous studies conducted outside of stroke populations, using serum samples instead of plasma, or in comparison to other lab-based GFAP assays.

The objective of this proof-of-concept analysis was to evaluate the analytical agreement between the Upfront DX LVOne GFAP LFA in comparison to a gold-standard lab-based assay [Single Molecule Arrays (Simoa®)-based Human Neurology 4-Plex B assay (Quanterix Corp.)] using samples from TBI patients and healthy volunteers. Specifically, the study aims to assess the ability of the LVOne GFAP LFA for the identification of samples with GFAP concentrations above the manufacturer’s reported lower limit of detection (≥ 0.2 ng/ml) alongside the association between the LVOne GFAP LFT semiquantitative values with those obtained from the gold-standard assay.

## Methodology

### Selection of Samples

Fifty serum samples were selected from a local TBI biorepository comprising of adult TBI patients and healthy adult volunteers, collected as part of a prospective observational study of TBI patients admitted to neurointensive care, conducted between September 2021 and March 2022. The recruitment criteria for TBI patients in this study were adults (aged > 18), with no prior significant neurological disease, admitted to the Neurosciences and Trauma Critical Care Unit of a Major Trauma Centre (Addenbrooke’s Hospital, UK) following head injury with a clinical diagnosis of TBI. All patients required intubation and ventilation for management of their injuries and received protocolised intracranial pressure management. Healthy volunteers were all adults (aged > 18) with no prior history of TBI or neurological disease. Each TBI patient had multiple blood samples taken at various time points throughout their neurocritical care admission. The samples used in this analysis were selected based on reported GFAP concentrations as quantified by the Simoa® assay. Sample selection was designed to include a range of GFAP levels in order to evaluate the dynamic range of the LVOne GFAP LFA, with particular emphasis on concentrations near the assay’s lower limit of detection. A convenience sample was used, and the sample size (*n* = 50) was limited by the number of LVOne LFT devices made available from the manufacturer.

Ethical approval was obtained from the Cambridgeshire 2 Local Research Ethics Committee (LREC 97/290) and written informed consent, or written assent from the next-of-kin where appropriate, was obtained in all cases. Clinical variables for the TBI patients were extracted from the electronic clinical record. Radiological variables were extracted from the clinical report of the first brain CT performed in the tertiary centre, with variables extracted in accordance with the National Institute of Neurological Disorders and Stroke (NINDS) Common Data Elements (CDE’s) for TBI (Vande Vyvere et al. 2024).

### Blood Collection and Quanterix Simoa® GFAP Quantification

Blood was collected into serum separator tubes either from indwelling vascular catheters (TBI patients) or from venepuncture (healthy volunteers) at Addenbrookes Hospital, Cambridge, United Kingdom. Samples were centrifuged at 1200× *g* for 10 min, aliquoted and stored at − 80 °C prior to analysis at the University of Cambridge. For serum GFAP quantification by Simoa®, samples were thawed and analysed using Quanterix Simoa® HD-X Human Neurology 4-Plex B assay (Quanterix Corp.) according to the manufacturer’s instructions.

### LVOne Lateral Flow Test

The selected serum samples were thawed for 30 min at room temperature and then analysed using the Upfront DX LVOne GFAP LFT according to manufacturer’s instructions. Briefly, 10 µL of sample were added to the test’s sample port, followed by addition of 100 µL proprietary chase buffer. The buffer was stored refrigerated, and no reagents required freezing. No additional preparation steps were necessary. Tests were incubated for 15 min before the test line intensities on the LVOne test were compared against a colorimetric reference chart by two blinded independent observers, with concordant measurements recorded. When there was discordance between the two observers, the test result was re-examined jointly, and a consensus reached on the final value to be recorded. Scores could range between 1 and 12 based upon the intensity of the test line. A test was considered as “positive” if a test line intensity ≥ 1 was present (1–12), and negative if there was no test line.

### Statistical Analysis

Demographics and clinical details were summarised using descriptive statistics with medians (IQR) for continuous variables and count (%) for categorical variables. The sensitivity and specificity for the LVOne GFAP LFA was calculated based on a contingency matrix built on the presence of a Simoa® GFAP concentration above or below the manufacturers reported lower limit of detection (0.2 ng/ml). The median, IQR and range of Simoa® quantified GFAP per LVOne GFAP LFT semiquantitative score was calculated. Correlation between the semiquantitative LVOne score and the Quanterix GFAP level was made using Spearman’s Rank correlation co-efficient. Due to the inclusion of repeated sample results from the same subjects, a linear-mixed-effects model was fitted to further examine the association between the LVOne semiquantitative score and the Quanterix-measured GFAP level. A random intercept was included for each subject to account for within-subject correlation, with the Quanterix-measured GFAP level used as the dependent variable. Model assumptions were assessed using visual inspection of the residual plots.

The study was conducted and written in accordance with the standards for reporting diagnostic accuracy (STARD) (Supplementary Appendix) (Bossuyt et al. 2015).

## Results

### Demographics and Sample Distribution of the Cohort

**Fig. 1:**
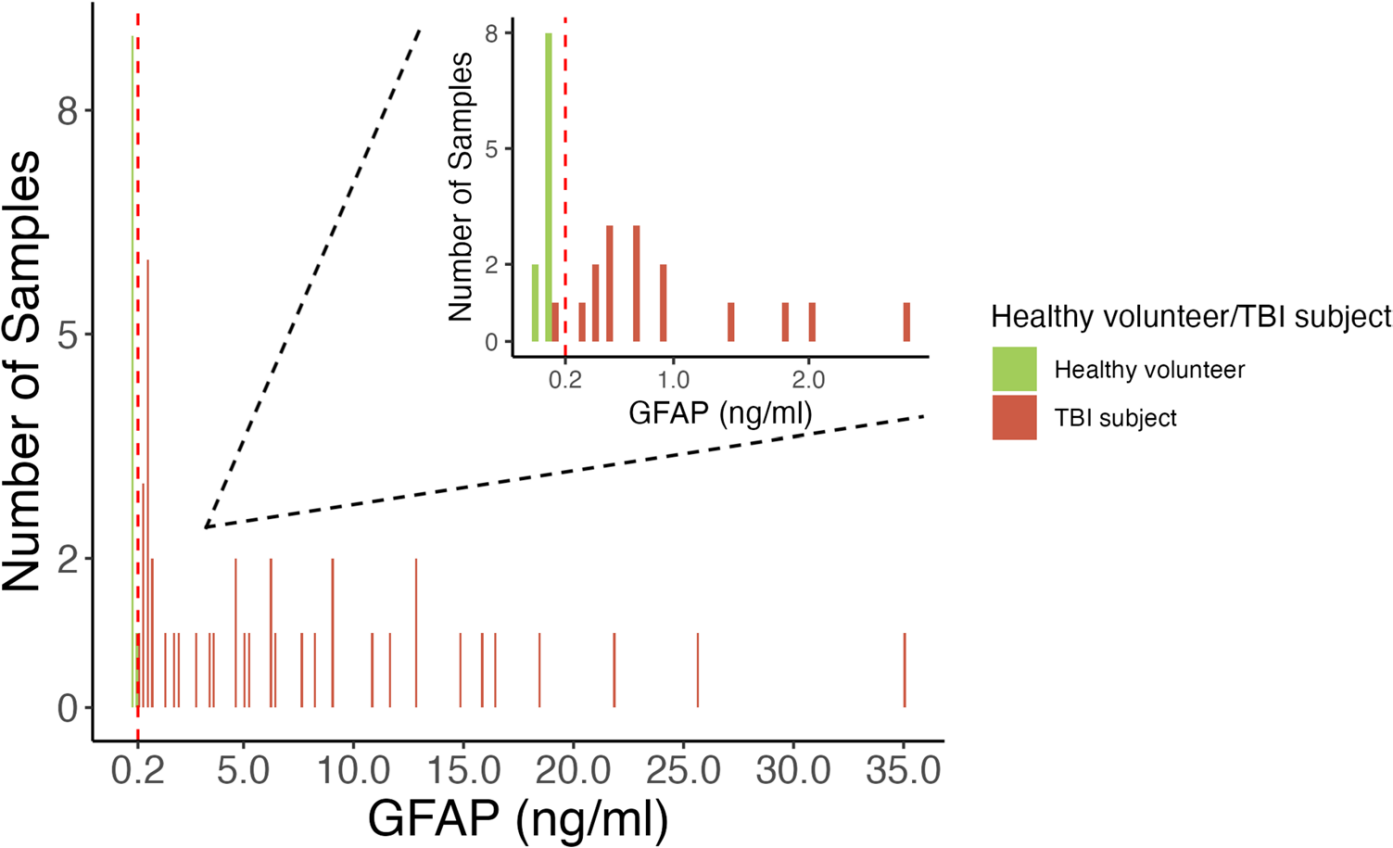
The distribution of GFAP levels (measured using the Quanterix Simoa® Human Neurology 4-Plex B assay) of serum samples taken from TBI patients and healthy volunteers. Nested chart demonstrating the distribution of samples with GFAP < 5 ng/ml around the lower limit of detection (0.2 ng/ml) for the Upfront Dx LVOne GFAP lateral flow assay. Red dashed line at the lower limit of detection for the LVOne GFAP lateral flow assay (GFAP = 0.2 ng/ml). *GFAP* glial fibrillary acidic protein

**Fig. 2:**
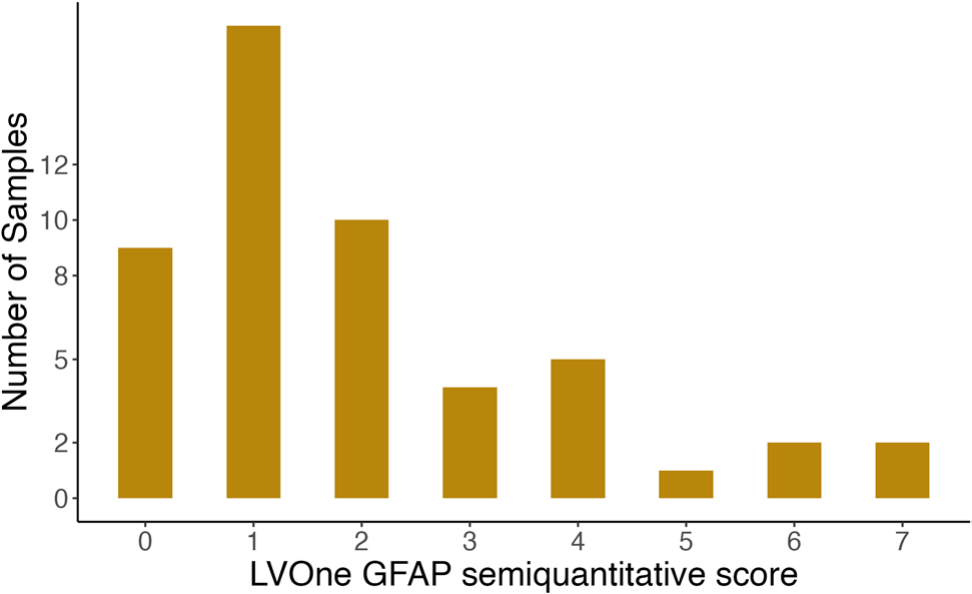
The distribution of Upfront Dx LVOne GFAP lateral flow assay semiquantitative scores. *GFAP* glial fibrillary acidic protein

The median (IQR) age of all participants was 34 years (IQR: 24, 44) and 50% were male (healthy volunteers: age 25 years (IQR: 24, 34), 28.5% male; TBI patients: age 40 years (IQR: 27, 45), 60% male) (Table 1). Median post-resuscitation GCS in TBI patients was 6 (IQR: 4, 9; range: 3–15), with one patient having a GCS between 13 and 15 (mild), three having a GCS between 9 and 12 (moderate) and 11 having a GCS of 3–8 (severe). TBI patient samples were taken at a median of 53 h following injury, with a range of 2–331 h. Serum GFAP concentrations, as measured on the Quanterix Simoa® platform, ranged from 0.03 to 35.1 ng/ml, with 11 samples (10 healthy volunteers, 1 TBI sample) below the LVOne GFAP lower limit of detection for a positive GFAP test (Fig. 1). The distribution of LVOne GFAP semiquantitative results is demonstrated in Fig. 2.

**Table 1:** Demographics of participants

	TBI patients (*n* = 15)	Healthy volunteers (*n* = 7)
Age	40 [27.0, 45.5]	25 [23.5, 33.5]
Gender		
Male	9 (60%)	2 (29%)
Female	6 (40%)	5 (71%)
Pre-intubation GCS	6 [4, 9]	N/A
Injury severity		
Mild	1 (7%)	N/A
Moderate	3 20%)	N/A
Severe	11 (73%)	N/A
Any CT abnormality		
Yes	14 (93%)	N/A
CT		
IPH	8 (53%)	N/A
aSDH	9 (60%)	N/A
EDH	6 (40%)	N/A
TAI	3 (20%)	N/A
IVH	1 (7%)	N/A
tSAH	7 (47%)	N/A
Skull fracture	10 (67%)	N/A
MLS	3 (20%)	N/A
Mass effect	4 (27%)	N/A
Cisternal compression	4 (27%)	N/A
Extracranial injury (AIS ≥ 3)		
Yes	12 (80%)	N/A
Intracranial surgery		
Any	7 (47%)	N/A
Decompressive craniectomy	1 (7%)	N/A
Craniotomy	1 (7%)	N/A
Evacuation of SDH	2 (14%)	N/A
Evacuation of EDH	2 (14%)	N/A
Triple bolt insertion	1 (7%)	N/A
EVD insertion	1 (7%)	N/A
Time from injury to biomarker sample (hours)	52.8 [24.2, 110.4]	N/A
Day of sample following injury		
Day 1	10 (25%)	N/A
Day 1–7	24 (60%)	N/A
Day 7 onwards	6 (15%)	N/A

Median [IQR] of continous and number (%) of categorical variables

*GCS* glasgow coma score, *CT* computed tomography, *AIS* abreviated injury score, *aSDH* acute subdural haemorrhage, *tSAH* traumatic subarachnoid haemorrhage, *MLS* midline shift, *IPH* intraparenchymal haemorrhage, *IVH* intraventricular haemorrhage, *EDH* epidural haematoma, *TAI* traumatic axonal injury (indicated by multiple petechial haemorrhages)

### The Upfront LVOne GFAP Lateral Flow Assay Performance Around the Lower Limit of Detection

**Fig. 3:**
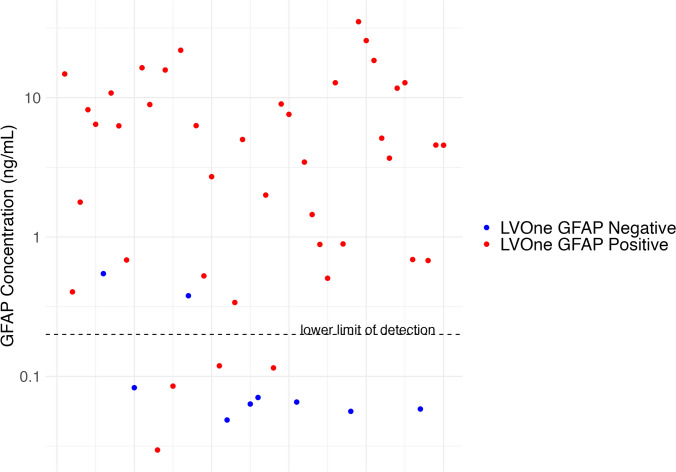
A comparison of the GFAP levels from the Upfront DX LVOne lateral flow assay (positive or negative) and the Quanterix Simoa® Human Neurology 4-Plex B assay. Each dot indicates a single test. Values of GFAP from the Quanterix Simoa® are shown in the logarithmic scale. Dashed line at the lower limit of detection for the LVOne GFAP LFA (GFAP = 0.2 ng/ml). Red/blue dots indicating LVOne GFAP positive or negative. *GFAP* glial fibrillary acidic protein

Of all samples, 41/50 had a positive LVOne GFAP LFT result, with 2 false negative and 4 false positive results. All false positives had a LVOne semiquantitative score of 1, with the 2 false negatives having Quanterix Simoa® GFAP levels of 0.378 and 0.546 ng/ml, respectively (Fig. 3). Overall the sensitivity of the LVOne GFAP LFA for a GFAP level ≥ 0.2 ng/ml was 95% (95% CI: 83%, 99%) with a specificity of 64% (95% CI: 31%, 89%), positive predictive value of 90% (95% CI: 77%, 97%) and a negative predictive value 78% (95% CI: 40%, 97%) (Table 2). The Quanterix Simoa® GFAP levels and LVOne semiquantitative scores of all false positives and negatives are displayed in Table 3.

**Table 2:** A 2 × 2 table of the Upfront Dx LVOne GFAP lateral flow assay results

	GFAP level > 0.2 ng/ml	GFAP level < 0.2 ng/ml	Total
LVOne GFAP + ve	37	4	41
LVOne GAFP − ve	2	7	9
Total	39	11	

*GFAP* glial fibrillary acidic protein

**Table 3:** False positives and false negatives of the Upfront Dx LVOne GFAP lateral flow assay

Participant number	Participant type	Quanterix Simoa® GFAP ng/ml	LVOne GFAP	LVOne GFAP semiquantitative score	CT findings
6	TBI	0.1190 (−)	Positive	1	aSDH
17	HV	0.0851 (−)	Positive	1	
17	HV	0.1150 (−)	Positive	1	
20	HV	0.0296 (−)	Positive	1	
14	TBI	0.379 (+)	Negative	0	EDH, Mass effect, Skull fracture
15	TBI	0.546 (+)	Negative	0	IPH, aSDH, Cisternal compression, Skull fracture

A positive LVOne GFAP test is indicated by a semiquantitative score of 1 or more

*GFAP* glial fibrillary acidic protein, *HV* healthy volunteer, *TBI* traumatic brain injury patient, *GCS* glasgow coma score, *CT* computed tomography, *aSDH* acute subdural haemorrhage, *IPH* intraparenchymal haemorrhage, *EDH* epidural haematoma

### The Association Between the Upfront Dx LVOne GFAP Lateral Flow Assay Test Semiquantitative Score and the Quanterix Simoa® Human Neurology 4-Plex B Assay GFAP Concentration

**Fig. 4:**
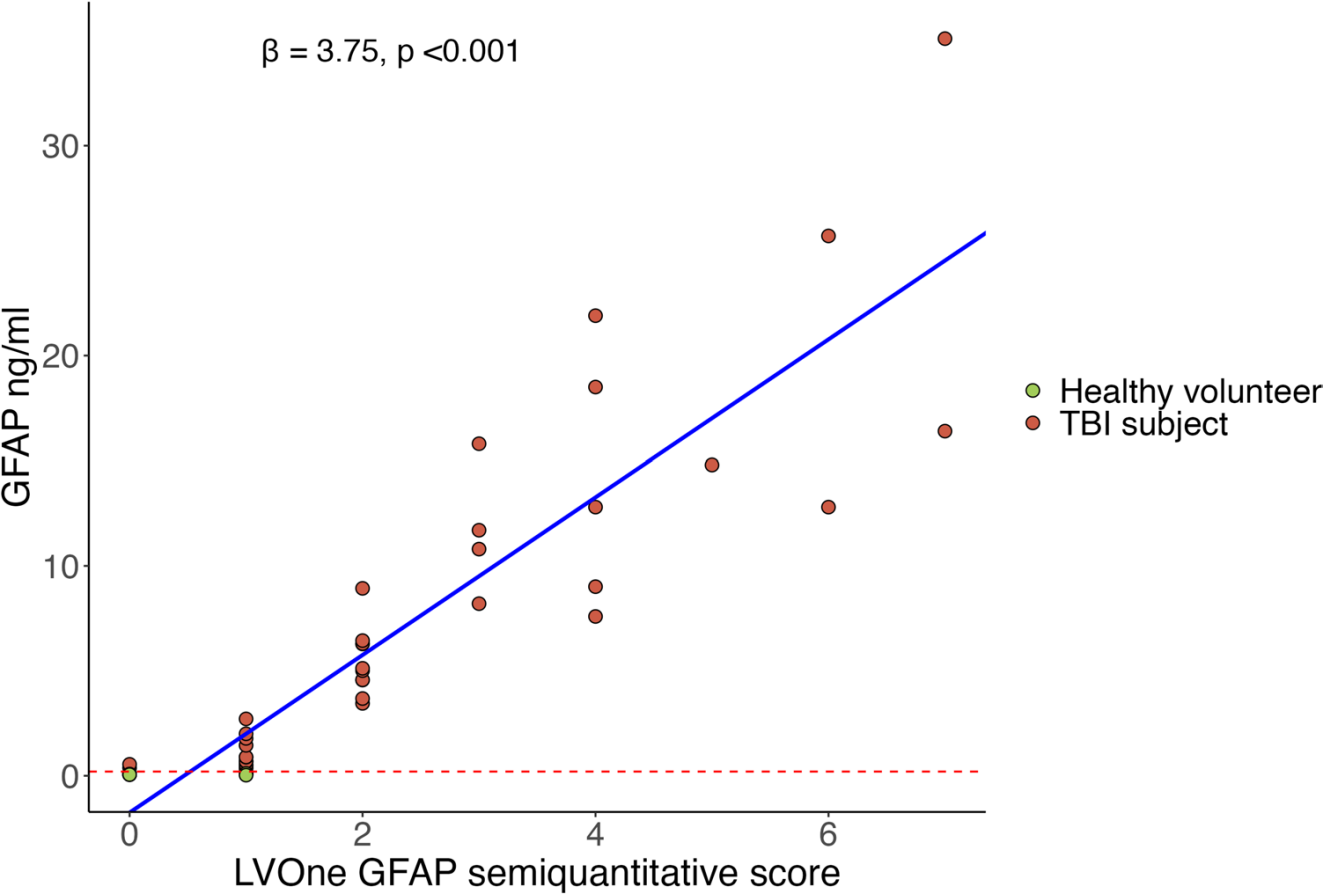
A scatterplot of the serum Quanterix Simoa® Human Neurology 4-Plex B assay GFAP concentration vs. the Upfront Dx LVOne GFAP lateral flow assay semiquantitative score. Red dashed line at the lower limit of detection value for the LVOne GFAP LFA (GFAP = 0.2 ng/ml). Regression line and co-efficient from a linear-mixed-effects model between Quanterix GFAP level and the semiquantitative LVOne GFAP score including the subject identifier as a random intercept. *GFAP* glial fibrillary acidic protein

The median GFAP, IQR and range for each LVOne semiquantitative score is provided in Table 4. There were fewer samples at higher semiquantitative scores, with large ranges of GFAP observed at each level. Overall, there was a significant positive correlation between the Quanterix-measured serum GFAP concentration and the LVOne GFAP semiquantitative score (*Rho* = 0.94, *p* < 0.001). A significant positive association was observed between the LVOne GFAP semiquantitative score and the Quanterix-measured GFAP concentrations, as determined using a linear-mixed-effects model accounting for subject-level variability [*β* = 3.75 (*SE* = 0.26), *t* = 14.21, *p* < 0.001]. This corresponds to an estimated increase of 3.75 ng/ml GFAP concentration for each one-level increase in the LVOne GFAP semiquantitative score (95% CI: 3.24–4.27) (Fig. 4).

**Table 4:** Median GFAP level (measured using Quanterix Simoa® Human Neurology 4-Plex B assay) per Upfront Dx LVOne GFAP lateral flow assay semiquantitative score

LVOne GFAP semiquantitative score	Number of samples	Quanterix Simoa® GFAP range (ng/ml)	Quanterix Simoa® GFAP (ng/ml) [median (IQR)]
0	9	0.049–0.546	0.065 [0.058, 0.083]
1	17	0.030–2.71	0.678 [0.339, 0.892]
2	10	3.45–8.93	5.065 [4.562, 6.295]
3	4	8.2–15.8	11.25 [10.15, 12.72]
4	5	7.59–21.9	12.8 [9.01, 18.5]
5	1	14.8	N/A
6	2	12.8–25.7	N/A
7	2	16.4–35.1	N/A

*GFAP* glial fibrillary acidic protein

## Discussion

In this technical assessment of a novel GFAP LFA in a cohort including samples from TBI patients and healthy volunteers, the Upfront Dx LVOne GFAP LFA demonstrated a high PPV (90%, 95% CI: 77%, 87%) and sensitivity (95%, 95% CI: 83%, 99%) in the detection of samples with a GFAP concentration exceeding the manufacturer’s reported lower limit of detection (0.2 ng/ml). Furthermore, there was a significant positive association between GFAP concentrations as measured using the Quanterix Simoa® Human Neurology 4-Plex B assay and the semiquantitative score provided by the LVOne GFAP LFT.

In a prior study, a different iteration of the LVOne GFAP LFA, employing a marginally higher threshold of GFAP (0.213 ng/ml) to indicate a positive test result, was used to assess plasma GFAP concentration in patients with suspected stroke. In this study, a significant positive correlation was observed between Abbott iSTAT TBI GFAP quantifications and the LVOne GFAP LFA qualitative results (*n* = 20, *Rho* = 0.86) with a single false positive and no false negatives (Gaude et al. 2025). These findings are largely consistent with the analysis presented in this study, which has extended the assessment of the LVOne GFAP LFA to a different disease state, used serum instead of plasma samples and compared it to a different lab-based GFAP assay. Overall, we observed a similar positive correlation between the semiquantitative LVOne GFAP test result and the lab quantified serum GFAP concentration. We did, however, observe a greater proportion of both false positives and false negatives than seen previously with the LVOne GFAP LFA. Of the false positives observed in our study, one was a TBI patient with an acute subdural haemorrahge on CT and three were healthy controls. The higher proportion of false positives and negatives in our analysis, in comparison to the prior study of the LVOne, may be due to the use of different iterations of the LVOne with marginally different GFAP thresholds and different test line intensities to indicate a positive LFT result.

Although they represented less than 5% of the overall sample, the false negatives identified in our analysis are of particular concern. Whilst multiple stages of further research and refinement are required before clinical use of the LVOne in a TBI population, it remains noteworthy that both patients with false negative results had intracranial injuries requiring neurosurgical intervention. If the LVOne LFT had been used to triage these patients, such results could have led to inappropriate conveyance and delays in care. Future research should incorporate clinical perspectives to define an acceptable threshold for false negatives and to establish what proportion of missed injuries or incorrect transfers can be tolerated. Whilst the answer to this will depend on the specific clinical application of the device, it remains a key question for future investigation.

Although prior, and ongoing, study of the LVOne GFAP LFT has focussed on LVO stroke, GFAP is a biomarker of considerable promise in TBI. Several large observational studies have found GFAP to be sensitive for the detection of traumatic pathology on brain CT (Bazarian et al. 2018; Czeiter et al. 2020), associate with the burden of parenchymal disease (Whitehouse et al. 2022), and be sensitive for the detection of CT occult structural damage later seen on acute MRI (Yue et al. 2019). Furthermore, of particular interest in relation to the potential clinical uses of a GFAP LFT, a hyperacute rise in GFAP has been demonstrated in a prehospital study where samples were collected within 30 min of sustaining a suspected moderate-to-severe TBI, with predictive ability demonstrated for the presence of CT pathology and the likelihood of requiring acute neurosurgical intervention (Papa et al. 2024).

It should be noted that the sampling performed in this study was for serum samples, which required laboratory preparation. Separate validation of the LVOne GFAP LFA will be necessary for the use of whole blood and/or capillary samples. However, although there are commercially available PoC platforms offering quantitative GFAP analysis of whole blood in a similar time-frame as the LVOne GFAP LFT (Kobeissy et al. 2024), should the LVOne, or similar tests, be validated for use with whole blood or capillary samples they may offer a distinct set of advantages which may complement or expand current testing options. Notably, LFTs do not require a dedicated analyser or cartridge-based system, which could reduce overall cost, minimise training requirements, and eliminate the need for ongoing calibration or maintenance. These features may enhance the device’s usability in resource-limited or austere clinical environments, such as pre-hospital settings, sports sidelines or low- and middle-income countries (LMICs), where infrastructure for conventional testing platforms may be lacking.

In the pre-hospital context, biomarker testing could facilitate more appropriate triage decisions, such as directing patients to neurosurgical versus non-neurosurgical centres, and allow for early activation of specialist teams, including neurosurgery, prior to arrival. (Tepas et al. 2013) It is therefore relevant that the LVOne test is currently undergoing prospective validation of capillary testing for GFAP and D-Dimer for the triaging of ischaemic stroke due to large vessel occlusion (ISRCTN12414986). Similarly, in LMICs where access to CT imaging is limited and may require long and expensive transfers, a robust, affordable device with minimal storage may assist with risk stratification and triaging decisions. Furthermore, biomarker testing at pitch side may allow for the identification of sports-related concussion following head impact, allowing appropriate withdrawal of players (O’Brien et al. 2025). Even within the ED, an LFT device may offer an efficient method for sampling of GFAP at the initial triage to direct care pathways and timely resource allocation. Overall, a GFAP LFT, such as the LVOne, could serve as a valuable adjunct or alternative to current available analysis platforms, particularly in settings where simplicity, portability, and robustness are paramount. However, significant future work and evidence, including both validation studies and research to prove clinical effect, are required prior to clinical use.

A notable limitation of the LVOne GFAP LFT is the subjective nature of the scoring against the colorimetric score card, which inherently lacks the precision of fully quantitative assays and may pose challenges for clinical interpretation. The requirement for inter-observer agreement in this analysis highlights this limitation and reflects potential variability in score assignment. Digital reading approaches have previously been trialled for a variety of LFT tests, and would present a useful adjunct to this technology, improving accuracy and clinical governance. For example, digital readers have been shown to have a higher accuracy than human-read LFT HIV tests in rural South Africa (Turbé et al. 2021), and for diagnosis of SARS-CoV-2 during the COVID-19 pandemic (UK Health Security Agency 2022). A digital reader does not necessarily require a separate medical device, with smartphones commonly used to provide digital reading through custom made mobile phone applications (UK Health Security Agency 2022). A further limitation of the LVOne, outside of the controlled research environment, is the requirement to read the test at 15 min, with longer incubation times potentially invalidating the result. In time-pressured environments it may be difficult to strictly regulate time, and further investigation into a safe reading window may be required.

### Limitations

This study has additional limitations, the most notable being a retrospective analysis of a comparatively small sample size derived from a single disease state. The sample size was convenience based and limited by the availability of the LVOne LFTs, which leaves the study underpowered for true interrogation of the manufacturer reported lower limit of detection leading to large confidence intervals. Samples were selected based on the known quantified GFAP levels. Whilst this approach was intentionally designed to interrogate the LVOne GFAP LFA’s dynamic range, it may introduce selection bias and limit the generalisability of findings to an unselected clinical population. Ongoing prospective analysis of the LVOne is currently being conducted in a clinical stroke population. However, prior to clinical translation for different disease states, including TBI, further clinical evaluation is required.

The TBI patients studied were all admitted to neurocritical care, with the majority having a severe TBI. While this study was a technical examination of the LVOne GFAP LFA, it is important to note that the test may be of particular clinical value in the mild TBI population, and the lack of patients with mild TBI limits how far our results can be extrapolated clinically to this population. Therefore, targeted examination of the LVOne GFAP LFT within a mild TBI population is required to establish the utility and generalisability of this device. The lower limit of detection of the LVOne LFA (GFAP ≥ 0.2 ng/mL), as set by the manufacturer, is high in relation to the clinical thresholds of GFAP reported in the prior literature for TBI diagnosis or CT decision-making (Bazarian et al. 2018; Reyes et al. 2023; Papa et al. 2024). Although beyond the scope of this analysis, further assessment is required to determine the optimum cutoff of the LFA depending on the exact clinical use, with the lower limit of detection of the LVOne likely to require lowering for clinical diagnostic use in a TBI population. This may improve the accuracy of the test, particularly for mild TBI cases or regarding the diagnosis of sports-related concussion, where clinically important GFAP elevations are seen below the current detection threshold of the test (Reyes et al. 2023; Papa et al. 2024; O’Brien et al. 2025). The Quanterix Simoa® Human Neurology 4-Plex B assay is a research-use-only platform and is not intended for clinical diagnostic procedures. In this analysis, it was used as the reference comparator due to its analytical sensitivity, especially at low protein concentrations (Krausz et al. 2021), and widespread use in TBI research (Czeiter et al. 2020). Additionally, prior validation of the LVOne GFAP LFA has been conducted in a stroke population against a commercial PoC platform (Abbott iSTAT TBI Plasma), demonstrating a similar degree of correlation to that observed in our study (Gaude et al. 2025). However, future clinical validation of the LVOne in a TBI population should be undertaken in reference to clinically approved platforms.

There are multiple potential confounders of the acute GFAP level following TBI, including age, underlying comorbidities, and, critically, the timing of sample collection in relation to injury (Abdelhak et al. 2022). These factors can influence the interpretation of GFAP concentrations, and the thresholds considered clinically meaningful. However, the primary aim of this study was to compare the performance of the LVOne GFAP LFA with that of the gold-standard assay, focusing on analytical agreement rather than clinical interpretation. As such, biological and temporal determinants of GFAP expression, including sample timing, were not considered in this analysis. Future evaluations of the clinical utility of the LVOne will need to account for, and appropriately manage, these variables. Owing to the limited LVOne LFT availability, the samples were measured once without repeat testing, and therefore test–retest variability was not evaluated in this analysis. This represents a limitation and warrants further investigation in future studies. Finally, all LVOne LFT scoring was performed concurrently by observers, with a score only recorded following discussion, preventing the assessment of inter-observer reliability. Future evaluations of the LVOne should include independent observer scoring and formal assessment of inter-rater agreement.

## Conclusion

The Upfront DX LVOne GFAP LFA demonstrated good sensitivity and moderate specificity for detecting GFAP concentrations above 0.2 ng/mL in serum samples from TBI patients and healthy volunteers. Additionally, a significant positive association was observed between the LVOne GFAP LFT semiquantitative score and serum GFAP concentrations as measured by the Quanterix Simoa® assay. Replication of these findings in larger cohorts, particularly within mild TBI populations, is required, alongside assessment of use in real-world clinical situations with the inherent environmental and situational challenges this can bring.

## Supplementary Information

Below is the link to the electronic supplementary material.

Supplementary file1 (DOCX 35 KB)

## Data Availability

The datasets generated during and/or analysed during the current study are not publicly available but are available from the corresponding author on reasonable request.
